# Political enablers of ambitious climate policies: a framework and thematic review

**DOI:** 10.1038/s44168-024-00206-1

**Published:** 2025-02-14

**Authors:** Simon Montfort, Lukas Fesenfeld, Karin Ingold, William F. Lamb, Marina Andrijevic

**Affiliations:** 1 Oeschger Centre for Climate Change Research, University of Bern, Hochschulstrasse 4, Bern, 3012 Bern Switzerland; 2 Institute of Political Science, University of Bern, Fabrikstrasse 8, Bern, 3012 Bern Switzerland; 3 Human-Environment Relations in Urban Systems, École Polytechnique Fédérale de Lausanne, Station 2, Lausanne, 1015 Lausanne Switzerland; 4 Department of Humanities, Social, and Political Sciences, Eidgenössische Technische Hochschule Zürich, Haldeneggsteig 4, Zürich, 8006 Zürich Switzerland; 5 Department of Environmental Social Sciences, Eidgenössische Anstalt für Wasserversorgung, Abwasserreinigung und Gewässerschutz, Station 2, Dübendorf, 8600 Zürich Switzerland; 6 Mercator Research Institute on Global Commons and Climate Change, EUREF Campus 19, Berlin, 10829 Berlin Germany; 7 Potsdam Institute for Climate Impact Research (PIK), Member of the Leibniz Association, Telegrafenberg A 31, Potsdam, 14473 Brandenburg Germany; 8 Priestley Centre for Climate Futures, University of Leeds, Woodhouse, Leeds, LS2 9JT Leeds UK; 9 International Institute for Applied Systems Analysis, Schlossplatz 1, Laxenburg, A-2361 Nieder-Österreich Austria

**Keywords:** Climate-change policy, Climate-change mitigation, Climate-change mitigation

## Abstract

Currently, most research explaining why countries lead or lag in climate policy assumes a problem-oriented perspective, focusing on barriers to climate policy adoption. Here, we argue that correcting for past failures, solving problems, and bringing climate policies back on track for the Paris Agreement requires a solution-oriented perspective on the political enablers of ambitious climate policies. We unite a growing research community that has previously been scattered across disciplinary subfields with various ontological and epistemological assumptions. Rooted in a thematic review of the scientific literature, we introduce a framework with a typology of six political enablers for ambitious climate policy at its core. For each enabler, we summarize key policy implications. We illustrate our framework with a case study on the adoption of emission trading systems in the transport and building sectors in Germany and the European Union (EU) allowing future solution-oriented research to build on our effort.

## Introduction

The first Global Stocktake (GST), part of the institutional architecture to help track the progress on meeting the goals of the Paris Agreement, highlighted the rapidly closing window of opportunity for climate action in 2023^1^. National climate policies are still falling short of limiting global warming to the 1.5 °C temperature target pledged in the Paris Agreement^1^. Meanwhile climate change impacts are unfolding faster than initially predicted by scientists^2^. Reaching the target to triple renewables by 2030, as called for by the first GST^1^ and scientists by the Intergovernmental Panel on Climate Change (IPCC) [^3^, p. 22] as well as ensuring adequate updates of the nationally determined contributions (NDCs)^1^ requires a better understanding of the causal process around the political enablers that contribute to successful problem-solving in political processes. Broadly consistent with past definitions of political enablers, these include political factors that can be pragmatically altered in the near term to facilitate the adoption of increasingly ambitious climate policies at the country level^4^. Although structural, technological, geophysical, or economic factors may also act as enablers (for an overview, see ref. 5), understanding political enablers amenable to being pragmatically changed is crucial for countries to successfully “update and enhance” their pledges on climate action ahead of 2050 when net-zero greenhouse gas emissions should be reached.

Most existing theoretical frameworks that explain why countries lead or lag in climate policy assume a problem-oriented perspective that puts various barriers at the center of theorization and empirical analyses^6^. These perspectives advance our knowledge about factors hampering climate policy adoption^7–10^ or other forms of climate action^11^, primarily. Similarly, the IPCC synthesis reports cover barriers and specific mitigation options, such as renewable energy technologies[^12^, p. 144], but to a lesser extent the political enablers for overcoming barriers in political processes for ambitious climate change policy^13^. Many existing perspectives lack a causal process-based understanding of *how* political enablers contribute to problem-solving^14,15^ and crucial solution-oriented evidence remains scattered across disciplinary subfields in unstructured form, making the policy-relevant insights hard to access.

We call upon the scientific climate change research community, ranging from social sciences like political science to natural sciences, to address this important research gap. Specifically, we argue that it would be productive to incorporate more solution-oriented perspectives that focus on the specific causal pathways that enable problem-solving into theorization and empirical analyses. On this basis, we advance the literature by developing a framework based on a thematic synthesis of the existing knowledge on solutions into a typology of key political enablers for ambitious climate policy.

This typology—the core of our framework—unites insights previously scattered across disciplinary subfields with different ontological and epistemological assumptions, making crucial policy implications more easily accessible. For each type of enabler, we summarize the causal mechanisms for how it can solve problems in climate policymaking processes, and contextualize their applicability. Although not exhaustive, our typology provides a good overview of this literature and is rooted in a thematic review of 120 articles on key barriers to the adoption of climate policies. This will serve as an underpinning for a more solution-oriented future research agenda on climate policymaking. We illustrate our framework based on the empirical cases of the German national Emission Trading System (in German “Brennstoffemissionshandelsgesetz”; BEHG) and the EU Emission Trading System (ETS) II (ETS II) extension to transport and buildings. We selected these cases specifically as Germany and the EU are global climate leaders and thus “most likely cases”^16^, meaning that if we do not observe the enablers there, we are unlikely to observe them anywhere else.

## Results

### A Framework for enabling increasingly ambitious climate policies

The framework we advance here seeks to complement the predominantly problem-oriented literature^7–10,17^, which has focused on conceptualizing various barriers to ambitious climate policies. In contrast, our framework conceptualizes solution-oriented political enablers with the core assumption that these can remove or relax problem-oriented barriers inhibiting the adoption of more ambitious climate policies.

The theoretical framework provides an overview of a growing research community with various theoretical approaches and different ontological and epistemological traditions. It builds on research from political science, specifically, theories of the policy process from policy studies, insights from health sciences about evidence synthesis for the science-policy interface, psychological research on bounded rationality (i.e., behavior and perception biases), economists’ insights about the cost-effectiveness of policy instruments, and research from engineering related to renewable energy technologies. By doing so, the framework arrives at a condensed and integrated conceptualization of the political enablers previously scattered across these various theories and sub-fields of the literature.

Rooted in a historical institutionalist conception of policy change, the framework emphasizes actor preferences and political institutions, and how climate policies co-evolve endogenously^18^. The historical institutionalist conception emphasizes feedback effects undermining or reinforcing pre-existing developments depending on the specific design of the institutions and historical events. For instance, institutional arrangements and climate policy design, such as those from the political enablers, can foster coalition building later supporting more ambitious carbon pricing^17,19–21^.

**Fig. 1:**
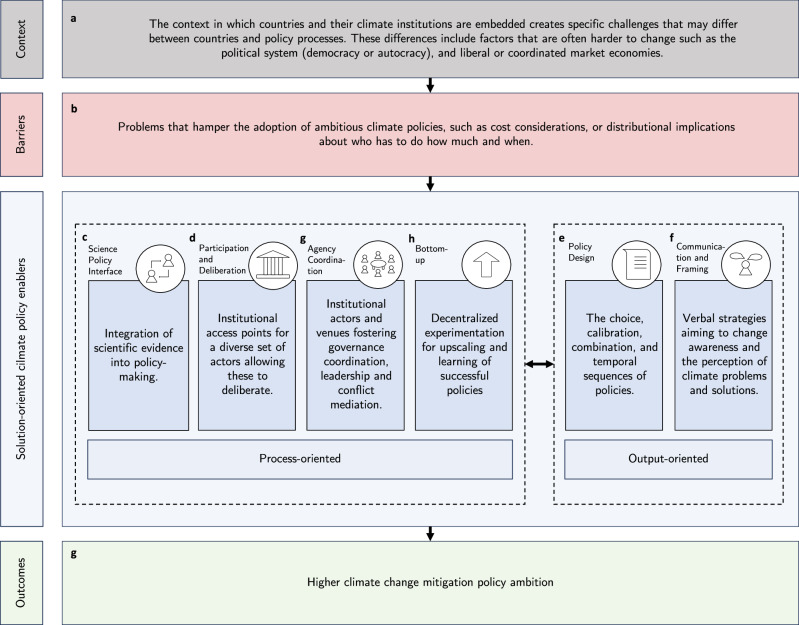
A solution-oriented framework of political enablers for higher climate change mitigation policy ambition. The key components include the context of the policy process (in panel **a**), the barriers (in **b**), the typology of political enablers (in **c**–**f**), and the outcome (in **g**).

The framework consists of four key elements: First, the context in which the political process exists includes rather rigid institutional factors that are hard to change, for instance, democratic or non-democratic checks and balances, such as electoral systems or the degree of centralization (Fig. 1a). External trigger events, such as environmental crises, are also part of the contextual factors and not of the barriers or political enablers as we conceptualize them here since they can arguably not be strategically changed to reach climate targets but are only used as “windows of opportunities” by policy entrepreneurs^22,23^. Second, it includes the barriers that have been relatively well conceptualized in earlier research^7–10,17^ (Fig. 1b, see also Table 1 for a summary). These represent the problems or obstacles to adopting climate policies in political processes, such as distributional struggles between actor groups about appropriate policies for reducing CO_2_ emissions. Third, the core of the framework lies in a typology of six enablers for more ambitious climate policies (Fig. 1c–f). Fourth, the framework includes political outcomes regarding the overall policy ambition or stringency level, which is key to achieving emission reductions under the Paris Agreement (Fig. 1g).

**Table 1:** Unpacking the enablers

Enabler		Specific enabling mechanisms	Example
	Science-Policy Interface	• informing about new solutions	• renewable^9^ and negative emission technologies^32^
		• monitoring	• emissions^142^ and low-carbon alternatives^143^
		• interface design	• inclusive scientific boards^30^
	Participation and Deliberation	• democratic processes	• pluralism promotes low-carbon interests^144^
		• new actor motivations	• reciprocity^36^, trust, cooperation^37^, consent^8^
		• stakeholder involvement	• civil society, consultation^41,42^
	Regulatory Agency Coordination	• vertical coordination	• top-down information exchange^50,51,59,145^
		• leadership	• brokers and entrepreneurs^53–55^
		• creation of institutional venues	• specific climate change institutions^35,47,49^
	Bottom-up	• local experimentation	• city-to-city networks^45^
		• up-scaling	• from local to national level^50^
		• carbon clubs	• carbon tariffs for non-compliance^146^
		• emission trading linkage	• unify fragmented markets^147^
	Policy Design	• instrument choice	• subsidies versus carbon pricing
		• instrument design	• carbon tax revenue recycling^73^
		• positive policy feedback	• benefit-inducing policies increase support^17,19,20,77^
		• policy packaging and co-benefits	• combining taxes and subsidies^69,70,89^
	Communication and Framing	• reducing partisan divisions	• decoupling identity politics^8^
		• emphasizing benefits	• raising awareness of benefits^105,148^
		• reframing	• employing positive wording^102^

Illustration of the typology with specific mechanisms that enable the relaxation of barriers to ambitious climate mitigation policies documented in the literature.

The typology includes six political enablers that can be leveraged strategically—a proposition validated in the empirical case study—to relax or circumvent barriers to increasingly ambitious climate policy. Four of these are predominantly policy-process-oriented, and two are mainly output-oriented. Process-oriented enablers include factors that may mainly be leveraged in political processes, for instance, interactions at the science-policy interface allowing for providing information about politically feasible policy designs. Output-oriented enablers emanate as concrete communication and policy outputs meaning that they focus predominantly on the results of the policy process. Both may interact and reinforce each other when present at the same time or in sequence. We develop definitions for each of these to structure future research. We then synthesize the insights from existing research around the causal pathways regarding how these may contribute to overcoming political barriers and enabling more ambitious climate policies.

#### Science–policy interface

The first of six broad categories of enablers that we introduce in this framework includes the science-policy interface. Research at the science–policy interface investigates the conditions for enabling the integration of scientific evidence into policy-making. Inputs into climate policymaking processes often start at the science-policy interface. Exchange at the science-policy interface can enhance the credibility of arguments in political processes and facilitate the integration of evidence into policymaking. Informing decision-makers about viable political pathways can reduce institutional capacity constraints, uncertainty, and information asymmetry.

On the scientific knowledge supply side, scientific advice at the science–policy interface is often preceded by systematic evidence synthesis. This can realign diverging roadmaps due to the plethora of studies from various scientific subdisciplines that reach different conclusions—sometimes called “cacophony”^24^ involving different opinions and evidence in scientific debates that often suggest diverging pathways to policymakers. Evidence synthesis is crucial for taking stock, explaining the system at hand, and resolving contradictory findings to arrive at robust conclusions about what works and why^25^. Using aggregated and robust evidence can facilitate access and uptake on the demand side by policymakers thanks to its credibility and often improved accessibility, allowing for the delegitimization of purely partisan-driven, opinionated interpretations of findings from single, potentially outlier studies^26^. If the supply and demand side of the science–policy interface align, they can effectively reduce institutional capacity constraints. This often alleviates information asymmetry and uncertainty about problem causes and solutions for decision-makers^27–29^. In doing so, the science–policy interface can play an important role in putting issues on the political agenda, monitoring target attainment, and advising on viable pathways for emission reductions in line with the targets.

Yet, prospects for an effective science-policy interface depend on the exact format, which may require tailoring to specific national or local settings, political institutions, traditions and cultures^30^. For instance, at the EU level, the Scientific Advisory Board on Climate Change consisting of 15 independent and interdisciplinary scientific experts informs about pathways for achieving the net zero targets set in the European Climate Law for specific policy instruments, such as the emission trading system. These include experts from several member countries and a range of scientific disciplines for a balanced representation.

The science–policy interface can also provide direct advice on policies and technological innovations, which in turn can lead to a ratcheting-up of climate policy ambition^9,23,31–34^. Expert panels can, for instance, explain the interaction between emission trading systems and other policies^31^. Scientific communities can create awareness by highlighting the opportunities and risks associated with technological and behavioral mitigation options^9^, for instance, through so-called epistemic communities – groups of actors acknowledged as experts who share beliefs about the value of specific mitigation options. Such experts and epistemic communities can act as brokers and policy entrepreneurs^23^ by contributing to the broadening^32^ and prioritization of technologically and politically more feasible policy options^9,33^. This can also reduce economic costs and distributional barriers to climate mitigation policies.

#### Participation and deliberation

Second, we identify participation and deliberation as factors that can enable more ambitious climate policy. Stakeholder participation creates access points for a diverse set of actors allowing for consultation of and deliberation about different views. Participatory and deliberative processes can take several forms and enable the adoption of more ambitious climate policies. These include civil society involvement, consultation of stakeholder positions, commissions and deliberative assemblies where either stakeholders^35^ or citizens make proposals to the government^36^. Participatory voice options create access points for diverse actors, including citizens, organized civil society actors, and firms. Including such diverse stakeholders in climate change governance can increase legitimacy, reciprocity^36^, trust, and cooperation aimed at solving collective action problems^37^. Different forms of deliberative democracy, such as citizen assemblies^8,36^ that allow citizens to participate actively can also reduce polarization and ensure that more diverse opinions are considered in decision-making processes. This can relax distributional barriers by increasing public support^38^.

Opening up policy processes also creates new forms of peer accountability that allow a more diverse set of actors^39^ to observe actions, pose questions, and sanction misbehavior, albeit often informally. This can increase the problem-solving and planning capacity of governments while countering the influence of more entrenched actors that favor the status quo^40^. In less democratic countries with lower levels of democratic input legitimacy, civil society involvement can increase support for climate policy, despite the potential lack of independence of the latter^41^. Similar results are found for democratic countries^41,42^.

Other scholars, however, highlight that the greater the number of actors with more diverse interests in policy processes, the more veto players who may block decisions, stall progress, and prolong decision-making procedures^43^. Depending on the format, deliberative and participatory process may also lower the democratic input legitimacy as non-elected actors can participate and citizens lack a voice option. Here, the specific form and design of the participatory, deliberative process, namely the fit and integration of participatory and deliberative processes into the broader institutional setting, are essential for its success. Transparent participatory and deliberative processes may turn out to be more effective in increasing policy ambition if they open up the policy process to other actors than special interest groups who are often well-organized^44^.

#### Regulatory agency coordination

The third enabling factor for more ambitious climate policy is regulatory agency coordination. This enabler includes institutional actors and venues fostering the coordination between governing agencies, ensuring entrepreneurship, and reconciling conflicting parties. Regulatory agencies can coordinate internally and externally with other governmental agencies and non-governmental actors in policy processes^45,46^.

Internal coordination includes vertical and horizontal structures that reduce information asymmetry within state institutions. Internal coordination can broaden the range of available policies by facilitating the cross-sectoral integration of successful policy options^47–49^. For instance, de Oliveira (p. 1906)^50^ describes how some institutions that addressed air pollution and climate change enriched available policy options through cross-sectoral knowledge transfer for policy implementation in the climate change domain. Thus, coordination between government agencies can buttress administrative capacity^50,51^, especially when important policy entrepreneurs and leaders can link new policy design ideas^51,52^.

External coordination structures relate to the government’s proactive management of policy processes. Evidence on external coordination at the international governance level has shown that leadership and conflict mediation through a so-called “lediator” strategy was crucial in negotiations before the landmark signing of the Paris Agreement by the national delegations of 194 sovereign nations^53^. It helped the organization of the summit, the structuring of the negotiations, and mediation between conflicting negotiation positions^54,55^. Besides the international realm, brokerage and conflict mediation by government agencies can also resolve conflict in national policy processes. For effective brokerage, stakeholders must perceive the broker as relatively neutral but as an influential leader in policy networks^56^.

#### Bottom-up processes

The fourth enabler for ambitious climate policies we identified centers around bottom-up processes. Research on bottom-up processes investigates conditions under which local, decentralized experimentation leads to upscaling and learning of successful policies that circumvent lacking global enforcement mechanisms to comply with international climate agreements. Bottom-up processes exhibit a relatively low degree of centralization that allows for experimentation with new governance arrangements and may trigger diffusion processes^57^, such as carbon clubs, in which cities or countries create horizontal governance arrangements for knowledge exchange^50,58–61^ or the establishment of a policy regime. Such policy regimes in the form of a carbon club include a coalition of countries that collaborate to promote and enforce climate policies, often by coordinating carbon pricing, trade incentives, and emissions standards to drive global climate action. Theoretical modelling has shown that club incentives, such as access to preferential trade agreements, financial support for green transitions, and shared technology, and the imposition of carbon tariffs or restrictions on non-members can encourage alignment with climate goals of such a club^61^.

To overcome the deadlock in international negotiations due to free-riding and distributional barriers, political action occurs bottom-up in decentralized national and subnational settings^19,57^. A sizeable body of research documents that local climate ambition can surpass national or international policy ambition (see, e.g., refs. ^58,60^). Such bottom-up processes may not only circumvent global free-riding barriers but also relax institutional capacity constraints. Constraints arising due to a lack of information or expertise can be relaxed via local experimentation and learning from other successful experiments leading to their diffusion via climate action networks, such as the Cities for Climate Protection (CCG) or ICLEI Local Governments for Sustainability^45,58^. This can also create bottom-up pressure^58^ that reinforces implementation capacity at the national^50^ or supranational level.

#### Policy design

The fifth enabler of more ambitious climate policy is policy design. This includes the choice, combination, calibration, and temporal sequences of policies. Research around this enabler investigates how the strategic choice of policy instruments^62–67^, such as taxes or subsidies, their combination^68–70^ and calibration^9,71^, for instance, related to carbon tax revenue recycling^72–76^, can enhance the political feasibility of ambitious climate policy.

From the economic efficiency perspective, carbon pricing policies are often favored over other options, such as low-carbon subsidies^73^ or reduced value-added tax rates^64^. However, efficient climate policies such as sufficiently high carbon prices often face high distributional barriers for citizens^73,77^ as well as vested fossil interests^78^. Such barriers can be addressed through policy sequencing meaning the strategic ordering of policies into benefit-to-cost sequences. First introducing theoretically second-best policies with lower acceptance hurdles, such as low-carbon technology subsidies, can foster innovation in renewables, reduce technology costs, create new opportunities to switch from fossil to renewable energies, and build supportive coalitions for more ambitious carbon pricing over time^17,19,20,77,79–81^.

Policy design not only has direct effects on technological innovation and human behavior^82^ but also influences ongoing political processes directly^73,75^ by shaping policy support and inducing policy feedback^77,81,83^. Such resource-based and interpretive policy feedback can affect the prospects of adopting increasingly ambitious climate policy over time^17,19,20,77,81,84–86^. Such feedback can also emanate from revenue recycling of carbon pricing for green spending to create new opportunity structures for low-emission behavior, such as electric vehicle charging stations^77^. A meta-regression of 100,000 respondents across 70 surveys in 26 countries found that green spending significantly boosts public support, possibly because many people do not fully understand the economic efficiency gains associated with equal redistribution of tax revenues to citizens, an option often advocated by economists as it minimizes market distortions with minimal administrative costs and creates distributional benefits for low-income households^76^.

Similarly, the literature on policy packaging^87,88^ suggests that non-market-based instruments, such as subsidies, may have complementary effects on the political feasibility of carbon pricing, especially when strong disruption to socio-technical and socioeconomic systems is expected due to job losses and stranded assets^69^. Coupling carbon pricing policies that impose direct costs on carbon emissions with policies that reward low-carbon technologies and behavior increases the feasibility of more ambitious carbon pricing^68,70,87,89,90^. Ex-post evaluations also suggest that combinations of policy instruments have been more successful in reducing emissions^91^. Research on climate policies in developing countries also increasingly emphasizes the role of co-benefits among development and climate change^72,92–94^. In many developing countries, climate finance is crucial for achieving emission reduction targets since large investments are necessary for transforming the economy and reducing economic cost barriers. One proposal is to make climate finance conditional on introducing carbon pricing. Revenues from carbon pricing may then be used to bolster low-carbon economic development. Although this may reduce economic cost barriers, it can lead to distributional trade-offs, for instance, for poor segments of society^72^. Compensation schemes may offset such regressive effects and enable just transitions^95^.

#### Communication and framing

Finally, communication and framing strategies can change the perception and awareness of climate change problems and solutions. Communication aims at creating awareness of climate change, political issues, and potential solutions. Framing can aim at increasing support for climate policy by changing the perception of the problem, its causes, and moral and normative conclusions about potential solutions^96^. Framing can take different forms, such as emphasizing specific arguments in political discourse, modifying the sender of a message (source cue), and changing the perceived (temporal, spatial, social) distance to a problem.

Political campaigns are often driven by distributional struggles rooted in partisan- and identity-based considerations, especially in two-party majoritarian political systems like the US. Consequently, the literature on communication and framing investigates to what extent such divisions can be overcome^8,97–100^. In contrast to most other enablers, communication and framing research centers mostly on resolving distributional barriers around who has to do how much and when. However, the evidence in this area is inconclusive about the extent to which framing strategies can overcome such divides. Some argue that decoupling communication from partisan- and identity-based considerations can positively affect support^8^ as a salient political identity negatively affects support^97^. Others point out that deep-rooted divisions are unlikely to be overcome only via communication and framing strategies^99^.

Closely related to this line of research is the framing literature investigating how changes in wording affect public support for climate mitigation policies^100,101^. This research finds, for instance, that framing climate change as an air pollution or energy security problem increases public support in the US among Republicans but not Democrats^100^. This means that framing interacts with other variables, such as political attitudes or prior knowledge, but tends to have relatively small effects by itself^102,103^.

Communication that raises awareness or involves talking about benefits rather than costs may potentially increase the feasibility of ambitious climate policy. For instance, consensus in parliamentary debates may emerge when opportunity-oriented discourses, rather than cost-based argumentation, realign positions^104^. Related research on communication with voters shows that the benefits of climate policies should be emphasized to increase support. Often, citizens know little about the benefits of climate-oriented political reforms^105^. For instance, regarding carbon taxation, it is essential to communicate progressive redistributive reimbursement mechanisms so that citizens perceive the policy benefits^65,73^.

### Case study: the combination and sequencing of enablers can become more than the sum of its parts

**Fig. 2:**
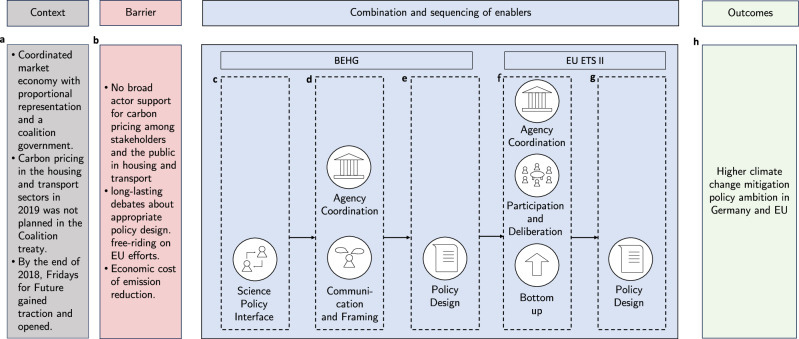
Case study illustration of the theoretical framework of political enablers. This illustrations shows the combination and sequencing of key solution-oriented enablers and how these fostered the introduction of the Fuel Emission Trading Act (BEHG) in Germany and the EU ETS II: the context (in panel **a**), the barriers (in **b**), the enablers (**c–g**), and the outcome (**h**).

We present a short case study (see Fig. 2) to exemplify the solution-oriented framework (see Fig. 1) of enablers for increasingly ambitious climate policy. We selected Germany and the EU as they are most likely cases^16^ since they are global climate leaders. Such most likely cases provide a first validation of a proposed theory rather than constituting a full-fledged theory test. Here, this case selection also offers the advantage that all key enablers can be observed empirically, thus illustrating how they operate in practice. Methodologically, we build on official government documents and secondary literature.

First, the German Brennstoffemissionshandelsgesetz (BEHG), or Fuel Emissions Trading Act, is Germany’s national emissions trading system, first adopted in 2019, and officially started in 2021. It covers CO_2_ emissions from the combustion of fossil fuels in sectors not included in the EU Emissions Trading System (EU ETS), specifically housing and transport. The BEHG sets a fixed carbon price for the initial phase, starting at €25 per ton of CO_2_ in 2021 and gradually increasing until 2025. After this phase, an auction-based pricing mechanism will be introduced. The system aims to incentivize fuel suppliers to reduce emissions through carbon pricing, ultimately encouraging a shift to cleaner fuels and energy efficiency in these sectors.

Second, building on the German BEHG, the EU ETS II is the European Union’s forthcoming extension of its existing Emissions Trading System (EU ETS I) to include emissions from the road transport and building sectors. Expected to be operational in 2027, the EU ETS II will apply a cap-and-trade mechanism similar to the original EU ETS. Emission trading systems use a cap on overall emission rights that is lower than what firms would emit in the absence of the policy. Firms may only emit CO2 if they hold a tradable permit. If a firm wants to pollute more, it needs to buy more permits while those firms who may cheaply reduce emissions find it more profitable to sell their emission permits. By including these additional sectors, namely transport and building, into the ETS system, the EU aims to provide a market-driven incentive for emission reductions, promoting greener technologies and practices in transport and housing. This extension is part of the EU’s broader strategy to reach its 2030 climate targets and achieve carbon neutrality by 2050.

#### Context

The policy process around the BEHG unfolded in the context of Germany as a coordinated market economy with a proportional representation (PR) voting system (Fig. 2a) embedded into the multi-level context of the EU. Coordinated market economies with PR favor “consensus” over particularistic interest groups in comparison to liberal market economies with first-past-the-post “majoritarian” voting systems^44^. Unless one party achieves the absolute majority, which is rarely the case, the parties with the most votes form a coalition government to achieve the majority^106^. This was also the case in September 2017 after the parliamentary election when the Christian Democrats/Christian Social Union (CDU/CSU) of re-elected Chancellor Angela Merkel formed a coalition government with the Social Democrats. In Germany, the coalition agreement typically outlines major planned policy changes. Although the coalition agreement between the Christian Democrats and Social Democrats in 2017 stated the objective of strengthening climate action, it did not foresee the introduction of a carbon price in the housing or transport sector^23,34^. The adoption of the BEHG can thus be considered an unexpected policy change.

#### Barriers

Three specific barriers to the adoption of carbon pricing in Germany and the EU’s housing and transport sector have been prominently discussed before policy adoption. These include distributional barriers related to a lack of political support for this policy, economic cost barriers due to the cost of decarbonization, and free-riding concerns if individual countries moved forward with emission pricing^17^ (Fig. 2b).

Distributional barriers have centered around deep-held convictions about the role of the state in regulating environmental pollution. This has led to long-standing debates about the choice of the most appropriate policy instrument for Germany and also at the EU level. While the left and the green parties typically favored subsidies and regulation, liberals preferred voluntary agreements^107^. This debate can be traced back to 1979 under the liberal government of Chancellor Helmut Kohl when three large state-owned utilities agreed to buy energy from renewable energy producers^108,109^. Later in the year 2000, the German red-green government under Chancellor Gerhard Schröder favored expanding subsidies through the expansion of a Feed-in-Tariff (FiT) over a carbon price to strengthen renewable power production^23,107,110^. The FiT ensured that renewable energy is purchased at a fixed price for a specified period.

Cost-related consideration of energy and climate policy has been another key barrier in German and EU debates throughout the last 30 years for both industrial producers as well as private households^111^. Powerful producer groups have long opposed ambitious carbon taxes with the argument that they would lead to higher production costs, lower investment incentives and potentially adverse effects on the international competitiveness of domestic firms^107^. As such, vested industrial interest groups have long opposed stringent climate policies. These interests have often been successful in lobbying the government^112^. However, in Germany, the Federation of German Industries (BDI), a powerful industry interest group, changed its position in 2018 arguing that, under certain conditions (e.g., an EU-wide carbon price), climate policy would not hurt the economy, despite previous concerns related to the competitiveness of the economy^113,114^.

Although Germany has often assumed leadership in European climate policy^115^, the relationship has not been always such. Climate mitigation efforts at the European level also create at least some degree of free-riding incentives for member states as supranational enforcement is lacking, or loopholes exist. In 2005, with the introduction of the EU ETS I, which covered emissions from the power production sector and energy-intensive industries, each EU member state had to specify an overall emissions cap and the allocation procedure of the tradable permits to firms. Allocation procedures had to be approved by the EU Commission. In the first phase of the EU ETS I from 2005-2008, a large overallocation occurred, due to industry lobbying due to vested interests^107^. This free-riding by German domestic industries on EU efforts was curbed in 2008 during the second phase of the EU ETS I when the EU Commission rejected an overgenerous allocation of tradable emission permits^107^. Similarly, ten years later, the EU adopted legally binding emission reduction targets to comply with the Paris Agreement emission reduction targets^113,114^. In case of non-compliance, member states would have had to pay a fine. This exerted significant pressure on Germany to reform its climate policies [^113^, p. 8].

In sum, distributional dynamics, economic cost, and free-riding^17^ presented significant barriers in the German and EU climate policy process before the adoption of the BEHG and EU ETSII.

#### Enablers

Before the adoption of the German BEHG in December 2019, several factors favorably influenced the course of the climate policy processes. Input about the cost-effective design started at the science–policy interface and was pushed by policy entrepreneurial actions of well-connected scientists^23,34^. Public discussions on climate change gained traction with the emergence of the Fridays for Future youth movements and street protests at the end of 2019. This opened a window of opportunity for science–policy entrepreneurs to invest significant efforts to build coalitions and provide expert advice to steer the public discourse in favor of the adoption of a cost-effective carbon price^23^. Reinforcing the position for several years, the Potsdam-Institut für Klimafolgenforschung (PIK), the Mercator Institute on Climate Change and the Global Commons (MCC)^116–118^, and the German Council of Economic Experts published an influential report just before the adoption of the BEHG. Workshops between scientists and key stakeholders were held, fostering exchange with government ministries and even Chancellor Merkel thanks to the high degree of legitimacy and credibility of the scientists. Chancellor Merkel, who was in regular exchanges with scientists, notably Ottmar Edenhofer, director of the MCC and PIK, assumed leadership and actively supported the BEHG^23^ (Fig. 2c).

Furthermore, the Agora Energiewende and the ÖKO Institute, two prominent think tanks, published a report on the social and distributional implications of carbon pricing in August 2019^119^. These reports allowed policymakers to echo scientific arguments and to back up their positions scientifically^23,34^. This contributed to positive communication and framing among the public and in the policy debate in favor of an emission trading system, highlighting the instruments’ efficiency and relatively low uncertainty for private actors (Fig. 2d).

Another important factor that enabled the adoption of the BEHG was its specific policy design. Based on scientific recommendations, the BEHG was designed as an emission trading system with a fixed price corridor. This design effectively worked as a carbon tax for the first years, however, it was technically an emission trading system that allowed a) for potential integration with existing or future EU-wide emission trading systems and b) for a public reframing that avoided the label “tax”. This was key to its political success because especially the Christian Democrats had promised their constituencies not to introduce any new taxes (Fig. 2e).

After the adoption of the German BEHG in the late fall of 2019, the same German coalition government under Merkel that introduced the BEHG in Germany also held the rotating EU presidency in the second half of the year 2020 when the EU Council decided that the EU should reduce emissions by 55% until 2030 in comparison to 1990^120^. The rotating presidency of the EU Council is tasked with fostering compromise and mediating conflict between the regulatory agencies in the political processes of the EU institutions (see Fig. 2f), notably the European Commission and Parliament^121,122^. Merkel was one of the oldest and most experienced country leaders in the EU who was known for her compromise-seeking approach to policymaking and negotiation skills coupled with sophisticated expertize on policy problems and solutions^120^. During a debate in the German government, Merkel said: “By the end of the presidency, our aim is to have a unanimous decision by all member states that we agree on this 55% target for the EU” (cited in^123^). Achieving this was further facilitated through agency coordination, specifically the close interaction between Chancellor Merkel and EU Commission President von der Leyen, who was previously a minister in the Merkel government. When von der Leyen pushed her Green Deal agenda and the emission reduction targets, she needed effective, politically feasible, and concrete policy solutions to achieve these ambitious goals. The German BEHG offered such an example that already proved a workable policy solution in a major member state (Fig. 2e, g).

As such, the Merkel presidency of the EU not only took a decisive role in the brokering of and mediating in intergovernmental negotiations of the EU council but also in supporting a bottom-up transfer of the BEHG design to the EU ETS II (Fig. 2f). Formally proposed by the EU Commission, the executive organ of the EU, in the summer of 2021, the EU ETS II as part of the fit-for-55 package seeks to extend emission trading in the EU to buildings and transport - the same sectors covered by the German BEHG. In its core components, the design of the EU ETS II was inspired by the German BEHG^124^. Both the German BEHG and the EU ETS II follow a so-called upstream approach that increases the price on the supply side rather than pricing emissions on the demand side. However, the German BEHG has a broader scope covering all fuels and combustibles while the EU ETS II only covers those used in buildings and road transport^125^. Expert statements (interviewed in ref. 23) and survey data^126^ indicate that a potential bottom-up transfer was already part of the strategic considerations regarding the policy design and adoption process of the BEHG by leading German policymakers, science-policy entrepreneurs, and stakeholders (e.g., business associations). Therefore, for some of the enablers in our typology, the illustrative case study confirms existing insights from the literature showing that actors can strategically leverage political enablers, such as policy design (see e.g., ref. 17) to overcome key barriers. Our case study further confirms the role of active leadership and conflict mediation in regulatory agency coordination for successful bottom-up experimentation.

Although participatory and deliberative enablers (see Fig. 2f) arguably played a smaller role in the adoption process of the BEHG and EU ETSII, the policy design of the EU ETS II mirrors stakeholder support for the options that received the highest support in the consultation procedures. The EU-wide consultation procedures offer a way for diverse stakeholders to voice their opinions and serve to gauge the acceptance of policy design options for the emission trading system. This includes two main aspects: first, the introduction of a linear reduction factor successively reducing the emission cap over time was considered as important or very important by a total of 67% of all respondents. Second, a majority of respondents (53% in total) considered integration of the additional building and transport sectors to have a negative effect either because of insufficient price signals (18%), large differences between the sectors (19%), or potential disruptions of the stability of the EU ETS I (16%)^127^.

## Discussion: policy implications and future research

The GST exposed the critical need to adopt and implement more ambitious climate policies. Currently, most research that explains why countries lead or lag in climate policy assumes a problem-oriented perspective, focusing on the identification, and conceptualization of various barriers^6^. Pinpointing the problems is the right first step. However, correcting for past failures and bringing climate policies back on track for the Paris Agreement requires countries not only to identify political bottlenecks but also to pass through them. This implies the need for a solution-oriented perspective of climate policy research. Our typology of enablers advances a solution-oriented perspective by synthesizing the evidence that had previously been harder to access as it was scattered across the disciplinary and interdisciplinary literature. Our study has important policy implications for policymaking.

Creating access points for scientists to the policymaking process can increase decision-makers knowledge about potential solutions. This can be achieved by formally integrating scientific and expert advisory boards into climate laws^49^. Such legal clauses help to institutionalize the processes of the scientific monitoring of goal attainment providing the basis for scientific advice on the design of policies. Scientific evidence may help legitimize positions in policy processes by serving as a reference for credible information^128,129^, especially when backed by a supply of rigorous, replicable, and transparent evidence synthesis studies^24,130,131^.

Research on participation and deliberation highlights that policymaking processes should be opened up to include a diverse set of actors and limit the influence of vested interests that contribute to the sustained lock-in of fossil energies. This can contribute to leveling the playing field between civil society movements and vested interest groups. The latter typically have more financial means to organize, represent their interests, and veto ambitious climate policy when faced with more ambitious climate policy^41,42,44^. Broadening the input into policymaking may be achieved, for instance, through official government consultation procedures, as is being done, for example, in the EU or through citizen assemblies. Citizens can provide input into the policy process that is more representative of what people want^36^, especially when combined with leadership and organization from the science–policy interface.

To reconcile conflict and reduce the political polarization that often stalls policy change, climate policy processes should institutionalize conflict mediation structures that bolster so-called policy brokers who are perceived as relatively neutral in the policymaking process^54–56^. Furthermore, climate policymaking is often also hampered by fragmented governance, with climate-related tasks being divided across different regulatory agencies. To reduce sectoral entrenchment and account for cross-sectoral interdependencies, it is often advisable to set up dedicated climate agencies or coordination structures between pre-existing sectoral agencies. These agencies can facilitate the consideration of interdependencies between the sectoral approaches to climate policy for a holistic perspective on climate policy problems and solutions^50^.

Climate policy leaders, such as the EU and Germany, should seek to build so-called carbon clubs of various forms that facilitate information-sharing about local experiments with climate policies^57^ or that implement common policy schemes providing club members with an advantage over non-members. This can create incentives for other members to join. These carbon clubs, for instance, include city-to-city networks that unite subnational cities on climate action^50,59,60^. They also include carbon clubs between countries that impose taxes on carbon-intensive products and import taxes for non-members. Once a critical number of members join, becoming a club member becomes more attractive than staying out^61,132^.

Climate policy needs to generate visible and viable policy-induced benefits. Such policy-induced benefits can be introduced either at the same time through packaging cost- and benefit-inducing policies into one bundle^68,70,77,87,89^ or by redistributing revenues from a carbon tax to the population^73^. Combining benefits and costs can be done through policy packaging or the redistribution of tax revenues. As benefits such as the redistribution of revenues may remain unnoticed^133^, the latter is only likely to be successful if combined with careful communication to raise awareness of the former. Research on policy sequencing^17,19,20,77^, where benefits are introduced before carbon pricing, shows that the perception of benefits is a key driver of public support^77^. Moreover, such sequencing strategies can reduce left-right polarization in public support and, thus, reconcile conflictive positions in policy processes^77^.

Policymakers should be careful when communicating and framing the benefits of climate policy and emphasize the benefits to generate positive side effects and interactions with other enablers. Otherwise, benefits may often remain unnoticed^77,133^ or may be naturally given less weight due to loss aversion (which implies that humans value losses more negatively than gains of the same amount)^134^. Given the central importance of beliefs in public support, careful communication of the benefits should target the creation of positive beliefs about policies, for instance, related to fairness, effectiveness, and economic efficiency^135^.

Despite these practical implications, our framework also has limitations. Although we follow a transparent thematic reviewing methodology and reviewed 120 influential articles on key barriers to climate change mitigation policies to develop the typology of political enablers in our framework (see methods), it may be that more specific mechanisms within the broader groups of political enablers could be found. As our methodological procedure relies on selected articles covering barriers related to distributional dynamics, economic cost, institutional capacity, or multi-level free-riding, it may, in principle, be that more enablers could be identified from articles that discuss no or other barriers. Yet it is likely that papers on political enablers also name the barriers they address (and thus would most likely be captured in our search results). Another limitation of our thematic reviewing methodology is that the focus on the conceptualization preempts systematic insights about which of the enablers or what combinations of these political enablers are most effective in reducing barriers. Furthermore, the global research landscape of climate change is biased towards studies from the global north^131,136^, which is also the case for the literature underlying this framework. Thus, the framework is more applicable to Western developed democracies in comparison to developing countries.

Hence, future studies should enhance our understanding of individual enablers and their interactions across different contextual settings. This could involve quantitative or qualitative data to apply this framework in a detailed theory-testing set-up, using, for example, recently derived country-level climate policy stringency as the dependent variable^81,137–140^. Qualitative comparative research should extend beyond our illustrative example to investigate if and how different enablers function together, testing whether multiple enablers can synergize to foster the ratcheting up of climate policy ambition. Additionally, it would be valuable to gain deeper insights into the sequencing and combination of enablers that are most effective in varying contexts, such as democratic and autocratic countries, to understand better the scope conditions of political enablers in driving climate action.

## Methods

To construct the typology of political enablers, we employ established thematic synthesis methods^141^. Thematic synthesis strives for a qualitative synthesis of concepts, in our case the political enablers. For thematic synthesis, it may not be necessary to review all published papers as the synthesis and conceptualization of enablers typically do not fundamentally change after having reviewed a sufficient number of relevant articles (e.g., the conceptualization of an enabler does not change if twenty instead of five papers document the same enabler)^141^. In this sense, our approach is optimized for conceptual saturation of political enablers in social science studies that focus on established key barriers to ambitious climate policies across sectors. In line with the principles of this thematic synthesis method, we do not strive for exhaustive coverage^141^ as is the case in systematic maps^131,136^.

We inductively defined the typology of enablers based on an in-depth analysis of the existing literature on barriers to ambitious policy^17^. We organized our thematic review around four team workshops, in which we worked on the definitions and the causal mechanisms of the enablers to advance solution-oriented perspectives of political enablers to ambitious climate policy. In each workshop, the authors derived the enablers of ambitious climate policies for each of the four barriers introduced above, namely distributional dynamics, economic cost, institutional dynamics, and multi-level free riding introduced by ref. ^17^. To complete our thematic review, we employed a three-step methodological procedure:

In the first step, we identified relevant peer-reviewed articles on the four established key climate policy barriers, namely distributional dynamics, economic cost, institutional capacity, or multi-level free-riding^17^, from a machine-learning-assisted systematic map covering over 10,000 peer-reviewed research articles (see detailed methodology in ref. ^136^). We focused on these barriers because they have been relatively well conceptualized in previous research^17^, while enablers have not been equally well conceptualized and remained scattered across the literature. Furthermore, as we could not rely on an established framework of political enablers, we select articles focused on barriers because if an article discusses an enabler to overcome an existing barrier the article will typically also name the barrier. We chose articles predicted to contain a barrier to climate change mitigation policies from journals with the highest impact factors as these articles are influential and typically cited by many other articles. As such, the impact factor as a criterion for article selection allows capturing influential research that shapes current academic discourse, implying that many other articles build on the arguments in this research. The impact factor provides a forward-looking measure that takes into account the expected future citations. Therefore, by sampling articles based on the established conceptualization of key barriers^17^, we increase the chance of identifying and conceptualizing relevant enablers addressing these barriers. Our selected articles included a broad range of theoretical and empirical insights suitable to conceptualize the enablers of our typology consistent with the methods of thematic reviews^141^. In the second step, each workshop participant read the full text of the article and grouped key enablers. We then inductively defined our typology of important political enablers addressing the four barriers from 120 articles (30 for each of the four barriers). In the main text, we looked for sentences describing political enablers that can be pragmatically changed to relax barriers. We were particularly interested in the concepts of the enablers and the causal mechanisms, meaning the processes that may lead to the reduction of barriers. In the final step, we discussed the results, resolved inconsistencies, and merged our notes of specific enablers documented to relax barriers (see Supplementary Datasets 1–4, containing notes about the enablers mentioned in the literature). On this basis, the authors refined the definitions and the causal mechanisms that may lead to the reduction of barriers while drafting the article, yielding a comprehensive typology of climate policy enablers rooted in the existing social science literature.

We then illustrated our framework using an interdependent case study of the German Fuel Emission Trading Act (BEHG; ger.: “Brennstoffemissionshandelsgesetz”) and the EU Emission Trading System (ETS) II—two interdependent policy processes. We selected these two as they are most likely cases^16^ meaning that if we cannot trace the successful policy adoption as having been fostered by the enablers, we are unlikely to identify such influence else. To illustrate our framework in the case studies, we built on official government documents, including official reports, and secondary literature^23,34^.

## Supplementary information

Supplementary Dataset 1

Supplementary Dataset 2

Supplementary Dataset 3

Supplementary Dataset 4

## Data Availability

The data for the development of the typology is available in Supplementary Datasets 1–4.
